# Developing a simulation safety policy for translational simulation programs in healthcare

**DOI:** 10.1186/s41077-022-00200-9

**Published:** 2022-01-24

**Authors:** Victoria Brazil, Clare Scott, Jack Matulich, Brenton Shanahan

**Affiliations:** 1 Present Address: Faculty of Health Sciences and Medicine, Bond University, Gold Coast, QLD Australia; 2 Simulation Service, Nursing and Midwifery Research and Education Unit, Gold Coast Hospital and Health Service, 1 Hospital Boulevard, Southport, QLD Australia

**Keywords:** Simulation safety, In situ simulation

## Abstract

Healthcare simulation may present risks to safety, especially when delivered ‘in situ’—in real clinical environments—when lines between simulated and real practice may be blurred. We felt compelled to develop a simulation safety policy (SSP) after reading reports of adverse events in the healthcare simulation literature, editorials highlighting these safety risks, and reflecting on our own experience as a busy translational simulation service in a large healthcare institution.

The process for development of a comprehensive SSP for translational simulation programs is unclear. Personal correspondence with leaders of simulation programs like our own revealed a piecemeal approach in most institutions. In this article, we describe the process we used to develop the simulation safety policy at our health service, and crystalize principles that may provide guidance to simulation programs with similar challenges.

**Supplementary Information:**

The online version contains supplementary material available at 10.1186/s41077-022-00200-9.

## Background

Healthcare simulation is an established technique for improving patient safety, through training individual skills, teamwork behaviors, and by testing healthcare systems for latent safety threats. Paradoxically, we also know that healthcare simulation exercises may present risks to safety, especially when delivered ‘in situ’—in real clinical environments—when lines between simulated and real practice may be blurred. We felt compelled to develop a simulation safety policy (SSP) after reading reports of adverse events in the healthcare simulation literature, and editorials highlighting these safety risks [1]. We had experienced near misses in the first 5 years of operation of our simulation service and had developed ad hoc personal systems for safety that were inconsistent across our institution.

Achieving the goals of translational simulation [2]—directly targeting improvement in health service practice and outcomes—requires close physical proximity of simulated and real practice, and/or integration of simulation into real system processes (e.g., using hospital emergency call systems, or simulated patients listed in a hospital’s electronic medical record). System integrity may be disrupted—fake medications given to real patients [3], staff pre-occupied with treating a manikin when real patients require attention, or emergency call systems activated by mistake. More recently, large volumes of simulation training have been conducted to test “COVID-19 safe” care processes [4, 5], while ironically increasing the infection risks associated with gathering staff together for training, moving manikins between clinical spaces, and using personal protective equipment that may be in short supply for real patients [6].

Dan Raemer drew sharp attention to safety risks in an editorial jointly published by 3 of the leading health care simulation journals and offered the simulation community ‘ten commandments’ for safety [1]. His arguments are compelling, and we now need to provide practical guidance for practitioners working in translational simulation programs—where simulation is deliberately integrated and embedded in health service operations.

Managing safety risks associated with in situ simulation (ISS) is usually given a fleeting reference in the emerging academic conversation about the benefits of ISS, but there are few in depth explorations or empiric work. Bajaj and colleagues offer ‘No Go considerations’ for planned in situ simulation, to address the specific tension of staff and space resource allocation in a clinical environment [7]. Detailed suggestions for managing medication safety risks have been offered [8]. The paucity of literature on physical and system integrity risk is in sharp contrast to the volume of publications related to psychological safety risks in simulation.

The Society for Simulation in Healthcare (SSH) accreditation standards require “Mechanisms to protect and address physical and psychological safety of individuals involved in simulation, including orientation to the environment.” and “Mechanisms to appropriately separate simulation and actual patient care materials (e.g., equipment, supplies, and patient information).” [9]. The standards and companion document require “a policy to ensure separation of simulated and actual patient care supplies/equipment” [10], including labelling, management, cleaning, storage and disposal.

There are online resources available to help simulation providers translate these requirements into local context. The Foundation for Healthcare simulation Safety [11] offers links, resources, and printable medication label templates, and tools to support simulation safety briefings [12] are available online. These published resources are useful guidance for healthcare simulation safety but these need to be unified into a comprehensive approach within the local context of a simulation program. In particular, the volume and nature of in situ simulation delivery may need to be reflected in institutional approaches—what works in the ‘sim centre’ may not be adequate or appropriate for ISS.

Thus, despite the existence of standards and resources that encourage safety, the *process* for development of a comprehensive SSP for translational simulation services is not clear. Personal correspondence with leaders of simulation programs like our own revealed a piecemeal approach at most centres. In this article, we describe the process we used to develop the simulation safety policy at our institution and crystalize principles that may provide guidance to simulation programs with similar challenges.

## Context

The Simulation Service within the Gold Coast Hospital and Health Service (GCHHS) commenced operation in 2013, when the main hospital within the health service moved to a new physical facility. From the outset the simulation program was embedded as a *service* within the organization, with an overt mission of directly targeting health service improvement through a translational simulation approach [2]. This requires a large proportion of our simulation delivery occurring ‘in situ’ within clinical areas—to enable testing and improvement of systems, physical environments and to support authentic multidisciplinary team training. In 2020, 36% of our simulation delivery (344 h) occurred in clinical areas, including anaesthesia, birth suite, cardiac catheter laboratory, mental health, emergency department, operating theatres, outpatient clinics, neonatal intensive care, and pediatric, medical, and surgical wards. The Simulation Service also has available a modest suite of dedicated rooms for simulation delivery on the two hospital campuses with the health service where the service runs workshops and courses.

The GCHHS operates 2 major hospitals with 1150 beds combined and 4 community health centers. The health service employs over 8300 staff and has an annual operating budget over $AUD1.2 billion. The Simulation Service is staffed by four dedicated simulation educators with nursing professional backgrounds, supported by an assistant director of nursing and a medical director.

The Gold Coast Health Service has comprehensive policies and procedures for occupational health and safety and for supporting patient safety, and a well-defined process for the development, review and dissemination of any new policy.

## Policy development

Policies and procedures are mechanisms for planning, standardizing, and documenting the operations within health services [13]. They clarify organizational accountability, communicate consistent operating procedures to staff, and have become a central element of risk management within healthcare. Coherent policy frameworks are a requirement of accreditation for health services in most jurisdictions, e.g., the Australian Commission on Quality and Safety in Healthcare (ACQSHC) requires “..the organisation maintains a comprehensive set of organisational policies and associated procedures and protocols and reviews them regularly” [14].

Terminology in healthcare policy development lacks consensus, with “policy,” “procedure,” “guideline,” and “protocol” having variable definitions within specific national and institutional contexts. A policy broadly indicates the position and values of the organization, while protocols and procedures are more explicit and specific in detail [15]. Policies may relate to administrative, human resource management, care provision, medicines, and information management issues.

Effective policy development and management requires a consistent approach—how and by whom policies are drafted, review by affected stakeholders, authority for final approval, identified responsibility for communication and education, and policy maintenance and review [16]. The ACSQHC clinical governance standard outlines the policy processes, governance systems, and structures required to support policy development, implementation, monitoring, and evaluation [14].

Despite these requirements, published guidance on ‘best practice’ for healthcare policy development is limited, as is research on evaluation of effectiveness of hospital policies. Foley reported “inconsistency between what is expected by regulators, accreditors and managers and how hospital policy is actually enacted and practiced by frontline nurses.” [17]. Healthcare organizations frequently underestimate the barriers to implementation of policies and procedures [18].

Literature on the development of clinical practice guidelines is useful—guidelines should be relevant and useful for decision-making, transparent, overseen by a guideline development group, evidence informed, up-to-date, and accessible [19].

Within our Queensland state government context, there is a Department of Health Policy framework, with a Policy Management Policy and Policy Management Standard that specifies the governance of policy documents, and that “promotes a consistent and rigorous approach to policy development and approval, implementation.” [20].

The Gold Coast Simulation Safety Policy was developed over 18 months, in a process led by two of the simulation educators. Our final policy is available in Supplementary File 1, and permission has been granted to share this resource externally.

**Fig. 1:**
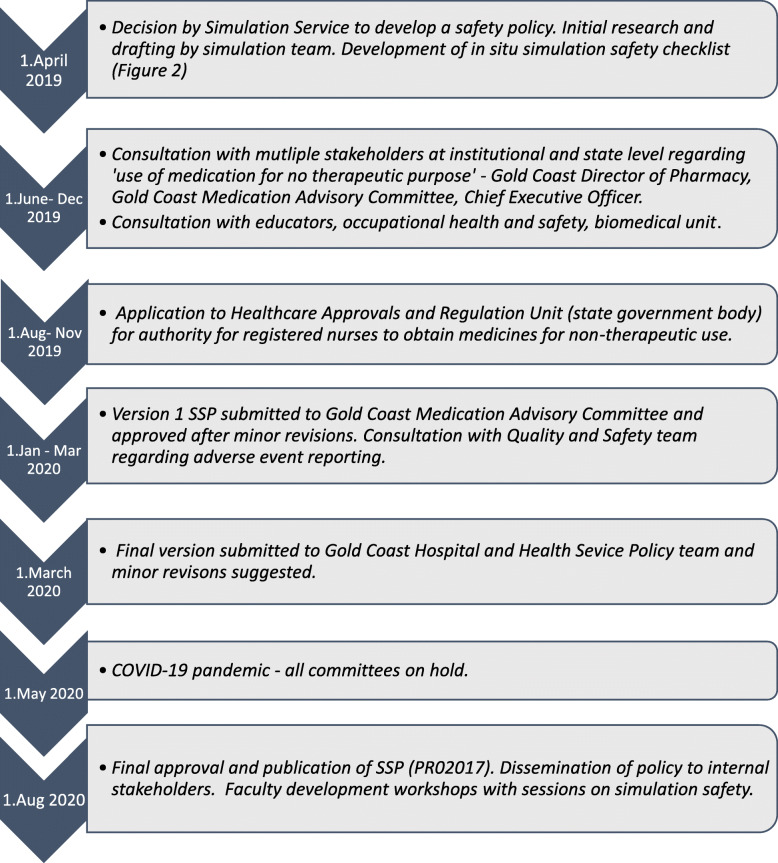
Timeline of Simulation Safety Policy development at Gold Coast Health

A timeline of our policy development process is offered in Fig. 1. It illustrates the importance of medication safety and associated regulatory requirements, and the extensive nature of consultations required within our health system context.

## Recommendations for simulation safety policy development

We offer the following practical steps for safety policy development, based on reflection on our experience, integrated with guidance from the literature.

1. *Form a STEERING GROUP for development and implementation of the simulation safety policy and identify RELEVANT STAKEHOLDERS required for advice and approval.*

Simulation activities that are closely integrated into the operations of a health service will impact on a broad range of stakeholders, including pharmacy, occupational health and safety, clinical departments, biomedical engineering unit, hospital switchboard, porterage staff. We had formal and informal consultations with these groups and recommend a balance of targeted stakeholder input with broader invitations across an institution. External advice (e.g., from device manufacturers) should also be sought where applicable. A review date should be determined prior to policy approval.

2. *Identify existing safety procedures for the health service/ educational institution that are relevant for the simulation program.*

Alignment and consistency with existing procedures is important, but challenging when health services are increasingly dynamic, and with hyperspecialized governance to match. Gold Coast Health has over 1200 procedure and policy documents covering over 220 services. Familiarity with niche policies saved invaluable time in drafting by establishing boundaries of scope and application, and early planning facilitated smoother integration of actions and accountabilities with existing safety systems.

3. *Incorporate simulation safety practices required in SSH accreditation processes and Raemer’s ‘Ten commandments’* *[*1*].*

Adapting generic resources to local context requires a granular approach. SSH has a template simulation policy [21] with a safety section (s19); however, much of the safety content is distributed throughout (s11 (psych), s20, s 22a, s23). Translational simulation programs will need to consider whether a single or separate policies are required for ‘center-based’ or in situ simulation activity. We opted for a single policy, with detailed content focused on ISS, based on our service profile.

4. *Consider the nature and extent of predicted safety risks, based on reports in the literature and local experience—adverse events and near misses.*

Our ‘near misses’ were mostly inadvertent emergency call system activation by clinicians deeply engaged in a scenario. Another near miss we experienced related to simulated patient (SP) physical safety, when an SP was forcefully physically restrained during a behavioural emergency simulation in which protective services officers were unaware of the encounter being a simulation exercise involving an actor. Colleagues in other institutions and literature reviews suggested medication safety was a priority.

After 5 years of operation, we had identified the moments in time, environmental situations and human scenarios in which risk was higher. For example, in a large scale trauma simulation exercise there is a risk that a simulated narcotic drug is used in real clinical practice, but risk is highest immediately following the simulation where it is left on the bench as participants move to the debriefing, or compounded in the event of a rapid exodus from the resuscitation bay to accommodate an emergent real patient arrival. Active reflection on these potential risks, even in the absence of any adverse events, and documentation in our regular simulation event reporting was useful in this risk assessment. This reflective process included post simulation debriefings with the simulation faculty teams and during regular Simulation Service meetings.

Engagement with quality and safety units may yield data and/or incidents from standard hospital reporting systems that illuminate risks not recognized by simulation educators. This may need more specific keyword style extraction if coding systems do not include simulation (such as ours). An additional strategy could include an anonymized survey to gain insight into areas people are less willing to talk about in person.

5. *Prioritize medication safety and liaise with health service pharmacy representatives*

Ensuring medication safety in simulated settings involves balancing risks [8]—bringing fake medications into real clinical areas versus using real drugs that may be subject to state or national legislation and have an associated cost. The benefits of translational simulation in identifying latent safety threats related to medication or testing new medication procedures are significant.

Our consultation process with pharmacy representatives was detailed and will be ongoing—as medication practices evolve, e.g., with the introduction of an electronic medical record, or changes to the contents of a resuscitation cart. We were grateful for existing work in this area, including the “Not for human Use” labels for medication, supplies and equipment as recommended by the Foundation for Simulation safety [11]

6. *Effectively communicate the existence of the simulation safety policy, and the need for staff involved in simulation delivery to comply with it.*

This was led by our core simulation delivery team and network of educators throughout the hospital. The existence of the policy was highlighted in simulation faculty development workshops, and available on the health service intranet, published with other policy and procedures. Electronic communication with departments planning translational simulation activities including copies of the policy and pointing recipients to relevant sections.

7. *Enable simulation faculty to conduct safe simulation sessions that are compliant with the policy, including structured briefings, cognitive aids and environmental cues.*

**Fig. 2:**
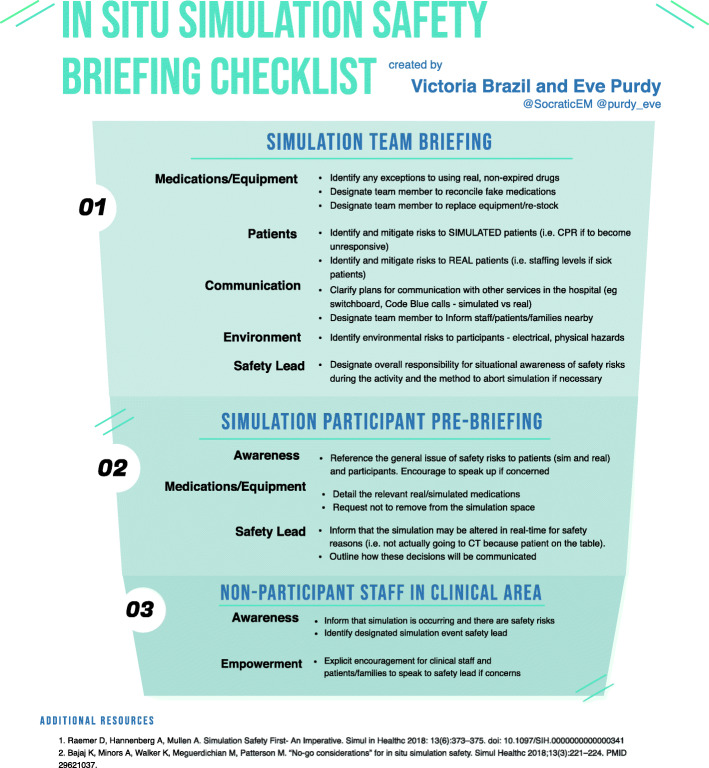
Checklist for simulation safety briefings

**Fig. 3:**
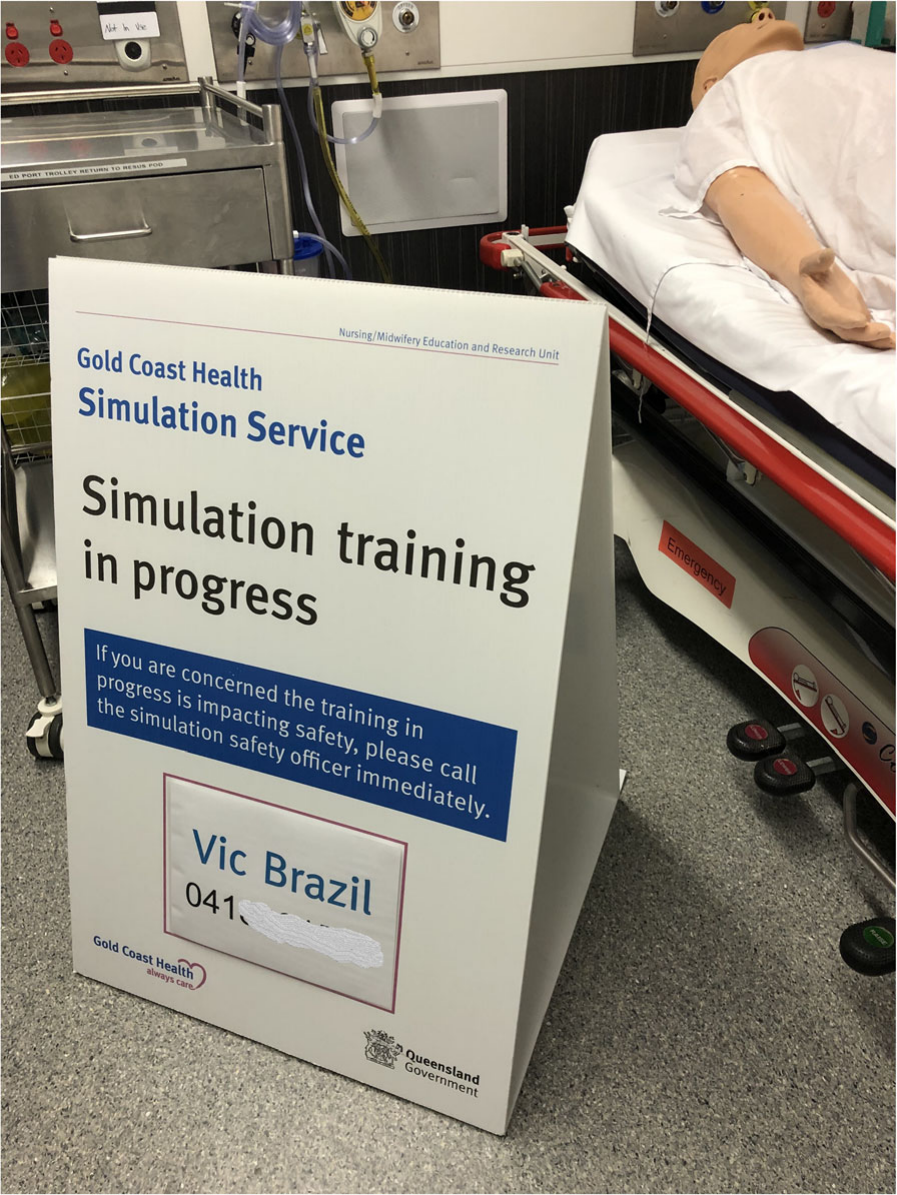
Example of simulation safety signage

We have developed a cognitive aid for simulation delivery teams conducting translational simulation activities (Fig. 2), as well as signage (Fig. 3) and staff uniforms to identify the simulation delivery team. A simulation safety officer is designated for each in situ simulation, and a safety briefing is incorporated into the overall simulation delivery team briefing and again during participant introductions and pre-briefing. These practical approaches were also integrated into faculty development programs.

Other examples of briefing tools and checklists are available on the Foundation for Healthcare Simulation Safety website [11].

8. *Develop a reporting process for simulation related adverse events or near misses, preferably integrated within the health service clinical adverse event reporting framework.*

In a fully integrated translational simulation service, a close relationship with quality and safety units allows rapid ‘sensing’ of risks in the clinical environment that are illuminated in simulation activities, as well as the opportunity to design simulation strategies directly targeting high risk practice areas. Reporting safety incidents related to simulation activity would be streamlined. We have not yet achieved a perfect connection in this regard, due to limitations with our online clinical reporting systems. However, we include any adverse events or high risk issues in our standard simulation event reports, which are circulated to relevant Quality and Safety leads.

## Conclusions

The nature and extent of risks associated with healthcare simulation delivery in clinical environments have been underappreciated, and the simulation community has inconsistent approaches to mitigate these.

Drawing on published guidance and our experience in developing an institutional simulation safety policy, we offer guidance for those seeking to develop a similar coherent approach to translational and in situ simulation safety in their own institutions.

## Supplementary Information

**Additional file1.** Infographic simulation safety policy.

## Data Availability

N/A.

## Abbreviations

SSP: Simulation Safety Policy
ISS: In situ simulation
SSH: Society for Simulation in Healthcare
SP: Simulated patient
ACQSHC: Australian Commission on Quality and Safety in Healthcare
