# Supplementary material for: Developing a simulation safety policy for translational simulation programs in healthcare
Source: Adv Simul (Lond). 2022 Jan 24;7:4. doi: 10.1186/s41077-022-00200-9 (PMC8785148; doi:10.1186/s41077-022-00200-9)
Supplement: Supplementary file 1 — Additional file1. Infographic simulation safety policy. [file 41077_2022_200_MOESM1_ESM.pdf]

DEVELOPING A SIMULATION SAFETY POLICY FOR TRANSLATIONAL SIMULATION PROGRAMS IN HEALTHCARE

V Brazil, C Scott, J Matulich, B Shanahan

Healthcare simulation may present risks to safety, especially when delivered 'in situ' – in real clinical environments - when lines between simulated and real practice may be blurred. These principles for developing a comprehensive simulation safety policy may provide guidance to simulation programs.

Form a STEERING GROUP for development and implementation of the safety policy and identify RELEVANT STAKEHOLDERS required for advice and approval.

Identify EXISTING SAFETY PROCEDURES for the health service/ educational institution in which the simulation program operates.

Incorporate safety practices required in SSIH ACCREDITATION processes and Raemer's 'TEN COMMANDMENTS'

Consider the nature and extent of PREDICTED SAFETY RISKS, based on reports in the literature and local experience – adverse events and near misses.

Prioritise MEDICATION SAFETY and liaise with health service pharmacy representatives

COMMUNICATE the existence of the safety policy and need for staff involved in simulation delivery to comply with it

ENABLE simulation faculty to conduct safe simulation sessions that are compliant with the policy, including STRUCTURED BRIEFINGS, COGNITIVE AIDS, and ENVIRONMENTAL CUES.

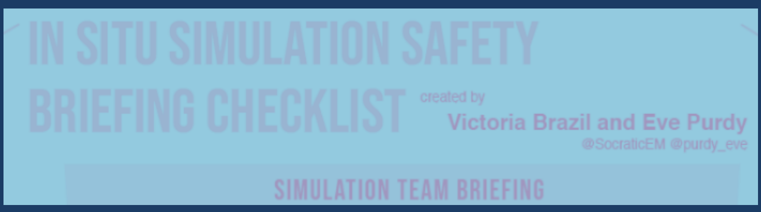

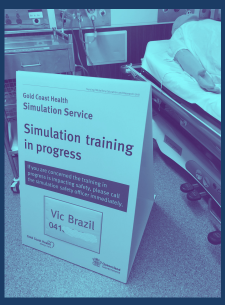

Develop a REPORTING PROCESS for simulation related adverse events or near misses, preferably integrated within the health service clinical adverse event reporting framework.

Queensland Government
Gold Coast Health

BOND UNIVERSITY
TRANSLATIONAL SIMULATION
